# Plasmatic Urea Nitrogen in Growing Rabbits with Different Combinations of Dietary Levels of Lysine, Sulphur Amino Acids and Threonine

**DOI:** 10.3390/ani10060946

**Published:** 2020-05-29

**Authors:** Pablo Jesús Marín-García, Mari Carmen López-Luján, Luís Ródenas, Eugenio Melchor Martínez-Paredes, Enrique Blas, Juan José Pascual

**Affiliations:** Institute for Animal Science and Technology, Universitat Politècnica de València, Camino de Vera s/n, 46022 Valencia, Spain; malolu@upv.es (M.C.L.-L.); luiromar@dca.upv.es (L.R.); eumarpa@upv.es (E.M.M.-P.); eblas@dca.upv.es (E.B.); jupascu@dca.upv.es (J.J.P.)

**Keywords:** growing rabbits, plasma urea nitrogen, amino acid, lysine, sulphur amino acid and threonine

## Abstract

**Simple Summary:**

Formulating diets to maximize nutrient harnessing has positive effects on performance and environment. In the case of growing rabbits, clues exist indicating that animals with high growth rate when consuming current diets show lower protein retention than expected, and it could be related to amino acid supply. The aim of this work is to find the amino acid combination (27 experimental diets: 3 levels of the 3 main limiting amino acids: lysine, sulphur amino acids, and threonine) that would minimize the nitrogen excretion in the bloodstream, a marker of the efficiency in the amino acid use This combination is a good candidate to be tested in order to improve performance and reduce pollution.

**Abstract:**

A total of 27 experimental diets were formulated starting from the same basal mixture, with a moderate content of crude protein and digestible energy (155 g and 9.86 MJ/kg of digestible matter (DM), respectively, both estimated). The contents of lysine, sulphur amino acids and threonine were variable. The first one, close to the current recommendations (Medium, M; 8.1, 5.8 and 6.9 g/kg DM for lysine, sulphur amino acids and threonine, respectively), and two other levels were on average 15% higher (High, H; 9.4, 6.6 and 7.8 g/kg DM for lysine, sulphur amino acids and threonine, respectively) or lower (Low, L; 6.7, 4.9 and 5.7 g/kg DM for lysine, sulphur amino acids and threonine, respectively). Diets were named with three letters, indicating lysine, sulphur amino acids and threonine levels, respectively. In total, 918 weaned rabbits (28 days old) were used (34 per diet). At weaning, animals were fed ad libitum with a commercial diet until day 46, day 47 each collective cage was randomly switched to one experimental diet. At day 48, blood samples were collected at 08:00h then the animals were subjected to 10 h of fasting and a second blood sample was extracted at 21.00h. At 08:00h, Pasmatic urea nitrogen (PUN) was higher with the L level of lysine (*p* < 0.001), unaffected by the level of sulphur amino acids and increased with the level of threonine (*p* < 0.001). At 21:00h, minimum PUN was observed with the MHL diet (14.72 ± 0.661 mg/dL). Taken into account the usual recommendations (established for a diet containing 11.3 MJ DE/kg DM, and then being 0.72, 0.51 and 0.61 g/MJ DE for lysine, sulphur amino acids and threonine, respectively), these results suggest that a diet containing more lysine and sulphur amino acids per energy unit (around 0.82 and 0.67 g/MJ DE) could better fit the growing rabbit requirements, although studies on the effects of such a diet on performance and protein retention are necessary.

## 1. Introduction

Selection for growth rate (with the aim to improve feed to gain ratio) has been done in paternal rabbit lines. However, some study suggests a loss in the effectiveness of this selection [1] which could be related to protein nutrition. Thus, with a diet containing 179 g CP/kg DM, [2] did not observed differences in relative growth, while when a diet with 161 g CP/kg DM was used, animals selected by growth rate had greater dissectible fat percentage and lower meat to bone ratio [3]. The authors of [4] showed that animals selected by growth rate had lower protein retention and higher energy retention than expected. On the other hand, as a consequence of the irruption of the epizootic rabbit enteropathy, dietary protein content has tended to be reduced nowadays [5]. In this context, the presence of some limiting amino acids could be suggested to explain the above commented findings.

The most frequently limiting amino acids are lysine, sulphur amino acids (methionine and cystine) and threonine. The requirements in these amino acids for growing rabbits are considered to be well-known [6]. However, these current recommendations have been established from dose-response studies, and in these studies the interaction between amino acids is not taken into account (i.e., it has been demonstrated in broilers that the requirements of glycine increases if dietary levels of methionine or arginine are low; [7]). Independently, most studies on amino acid requirements in growing rabbits were performed more than 25 years ago, so a review of these requirements seems necessary [8,9,10].

A large number of studies showed that low levels of plasmatic urea nitrogen (PUN), which correspond to the amount of nitrogen in form of urea circulating in the bloodstream, could be related with performance in pigs [11,12] or in broilers. Furthermore, [13,14] have demonstrated that PUN could be an indicator to detect amino acid deficiencies in diets for growing rabbits and have proposed an appropriate methodology to determine it. Furthermore, an oversupply is costly and leads to an excessive nitrogen excretion with a potentially negative environmental impact [15]. Knowing precise amino acid requirements could make it possible to develop different nutrition strategies with the aim to increase the biological value of the diet and reduce nitrogen excretion, improving the limitation of commonly use and non-environmentally friendly protein compounds in the rabbit diets.

The aim of this work was to evaluate PUN induced by the different dietary combinations of the three main limiting amino acids (using the current recommendations and increasing or decreasing them by 15%), in order to search for the combination minimizing PUN and, hypothetically, improving performance of rabbits with high growth rates. 

## 2. Material and Methods

The experimental procedure was approved by the Animal Welfare Ethics Committee of the Universitat Politècnica de València and carried out following the recommendations of the European Group on Rabbit Nutrition [16]. The experimental protocols followed the Spanish Royal Decree 53/2013 on the protection of animals used for scientific purposes [17].

### 2.1. Experimenal Diets

Twenty-seven experimental diets were formulated starting from the same basal mixture (Table 1). This basal mixture was formulated following the recommendations of all nutrients for growing rabbits [6], with a moderate content of CP (155 g/kg DM) and DE (9.86 MJ/kg DM). The contents of lysine, sulphur amino acids and threonine were variable (in a previous work, [4]) from the linear regression between protein retention and growth rate, we observed that protein retention in rabbit having very high growth rate was lower than expected, what would indicate some deficiency in amino acid supply. Then, we decided to compare PUN (a marker of the efficiency in the amino acid use) in rabbits with high growth rate fed with diets close to the current recommendations of the 3 main amino acids (medium), or having levels 15% higher (high) or 15% lower (low) of each. Thus, the main aim of this experiment is indicated in the title and expressed at the end of introduction section: “The aim of this work was to evaluate PUN induced by the different dietary combinations of the three main limiting amino acids (using the current recommendations and increasing or decreasing them by 15%), in order to search for the combination minimizing PUN and, hypothetically, improving performance of rabbits with high growth rates”, and three different levels were established for each of them (Table 2). The first was close to the current recommendations (Medium, M), and two other levels were on average 15% higher (High, H) or lower (Low, L). Diets were named with three letters, with the first, second and third letters indicating lysine, sulphur amino acids and threonine levels, respectively.

### 2.2. Animals and Experimental Procedures

One thousand one hundred and thirty-four weaned rabbits (28 days old) were allocated in 162 cages (6 batches, 27 cages/batch) of 7 animals. Among the animals that remained healthy at 46 days of age, 918 (34/diet) were selected and used for the experiment. Animals were from two different genetic lines in order to obtain a great variability in growth rate. These lines were line H (founded following a criterion of hyper-prolificacy at birth [18] and then selected by litter size at weaning over 17 generations, characterized by a large litter size at weaning but a standard growth rate) and line R [obtained after two generations of randomly mating from a pool of animals of three commercial paternal lines [19] and then selected by average daily gain during the growing period over 38 generations, characterized by a high growth rate].

Throughout the experimental period (April to December), animals were kept at 15 °C to 22 °C, with a photoperiod of 16 h of light and 8 h of darkness. At weaning, animals were identified, weighed and allocated to a collective cage of 7 animals (by distributing animals from every mother and line among different cages). Then, animals were fed ad libitum with a commercial feed (added with 35 ppm valnemulin and 250 ppm neomycin) for growing rabbits until 46 days old. At 08:00h on day 47, each collective cage was randomly switched to one of the 27 experimental diets, which was provided ad libitum. Following the methodology described by [14], at 08:00h on day 48 (after 24 h receiving the experimental diet), blood samples were taken from the central ear artery (1 mL in EDTA vials). Subsequently, the animals were subjected to 10 h of fasting and a second blood sample was extracted at 21:00h (3 h after refeeding). Blood samples were immediately centrifuged for 5 min at 700 G, and the supernatant plasma was frozen at −20 °C until further analysis. From this moment, animals were switched again to commercial feed provided ad libitum until 63 days old and then weighed to calculate the average daily gain during the growing period.

### 2.3. Chemical Analysis

The determination of PUN was performed using a commercial kit (Urea/BUN-Color, BioSystems S.A., Barcelona, Spain). The samples were defrosted and tempered, after which 1 μL was pipetted into test tubes (in each batch a standard and a blank were included). Later, 1 mL of reagent A (sodium salicylate 62 mmol/L, sodium nitroprusside 3.4 mmol/L, phosphate buffer 20 mmol/L and urease 500 U/mL) was added to each sample, mixed thoroughly and incubated for 5 min at 37 °C. Subsequently, 1 mL of reactant B (sodium hypochlorite 7 mmol/L and sodium hydroxide 150 mmol/L) was added, mixed thoroughly and incubated for other 5 min at 37 °C. Finally, the absorbance of each sample was read at 600 nm against the blank.

The amino acid content in diets was determined after acid hydrolysis with HCl 6N at 110 °C for 23 h as previously described [20] using a Waters HPLC system (Milford, MA, USA) consisting of two pumps (Mod. 515, Waters, Milford, MA, USA), an autosampler (Mod. 717, Waters, Milford, MA, USA), a fluorescence detector (Mod. 474, Waters, Milford, MA, USA) and a temperature control module. Aminobutyric acid was added as an internal standard after hydrolysis. The amino acids were derivatised with 6-aminoquinolyl-N-hydroxysuccinimidyl carbamate and separated with a C-18 reverse-phase column Waters Acc. Tag (150 mm × 3.9 mm). Methionine and cystine were determined separately as methionine sulphone and cysteic acid, respectively, after performic acid oxidation followed by acid hydrolysis.

### 2.4. Statistical Analysis

Data of PUN were fitted to a normal distribution. Although the samples were obtained by the same animals in both moments (08:00h and 21:00h), two independent analyses (separately for each sampling time: 08:00h and 21:00h) were performed (the main reasons for this separation are that the different feeding conditions, ad libitum vs. restriction, and caecotrophy’s interference provoked different physiological conditions and led to high variability both in the means and on standard errors, and a lack of homoscedasticity). In both cases, PUN (obtained of 918 animals) was analyzed as a variable dependent using a GLM model from the Statistical Analysis System [21], including the level of lysine, sulphur amino acid and threonine, all possible interactions (27 experimental diets) of these main effects, the batch effect and the average daily gain from 28 to 63 days old as covariates. Least square mean comparisons were performed by t-test. Furthermore, the representation of PUN obtained with each amino acid combination was fitted by linear regression using average daily gain as an independent variable with a regression (REG) procedure of SAS (SAS, 2009).

## 3. Results

Table 3 shows PUN values according to sampling time and the levels of the studied amino acids. The average PUN was highly dependent on the sampling conditions (mean ± standard error of mean of 11.47 ± 0.088 and 18.29 ± 0.152 mg/dL at 08:00 and 21:00h, respectively, *p* < 0.001). 

At 08:00h, PUN was higher with the L level of lysine (+1.08 ± 0.165 mg/dL, +9.7%, *p* < 0.001), unaffected by the level of sulphur amino acids and increased with the level of threonine. No significant effects of double or triple interactions and covariate (average daily gain) were detected (*p* > 0.2).

**Figure 1:**
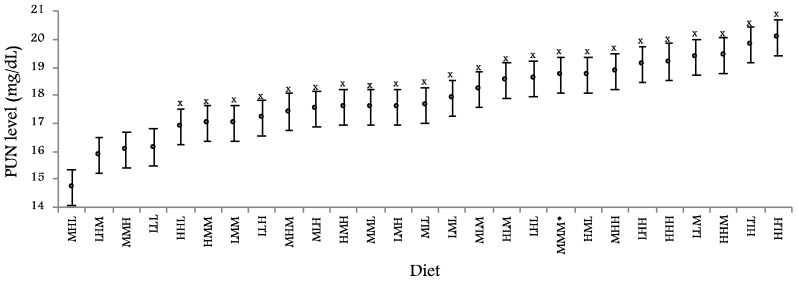
Plasmatic urea nitrogen (PUN) at 21:00h according to the experimental diet (*n* = between 29 and 34). First, second and third letters indicate lysine, sulphur amino acids and threonine levels, respectively (H: high; M: medium; L: low). Least square means ± standard errors. X value is significantly higher than the minimum, obtained with MHL diet (*p* < 0.05). * MMM: Is according current recommendations.

At 21:00h, PUN was higher with the H level of lysine (+1.05 ± 0.262 mg/dL, +6%, *p* < 0.001) and no significant effects of the levels of sulphur amino acids and threonine were observed. Nevertheless, interactions sulphur amino acids*threonine and lysine*sulphur amino acids*threonine as well as covariate (average daily gain) were significant (*p* < 0.001). Figure 1 shows PUN values obtained with the 27 experimental diets. Minimum PUN (14.72 ± 0.661 mg/dL) was observed with the MHL diet, although its difference with the 3 nearest values (LHM, MMH and LLL diets) was not significant (−1.29 ± 0.759 mg/dL, *p* = 0.090).

**Figure 2:**
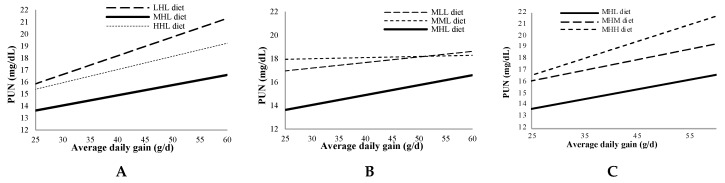
Relationship between plasmatic urea nitrogen (PUN) at 21:00h with average daily gain for MHL diet (bold line) compared with diets varying only in the level of one amino acid (**A**: lysine; **B**: sulphur amino acids; **C**: threonine). First, second and third letters indicate lysine, sulphur amino acids and threonine levels, respectively (H: high; M: medium; L: low).

The overall average daily gain was 44.1 ± 0.28 g/d (mean ± standard error). Figure 2 shows the relationship between PUN at 21:00h with the average daily gain for the MHL diet compared with diets varying only in the level of one amino acid. The average daily gain widely varied because two genetic lines were used, with lower values (25–35 g/d) being usual in healthy animals from maternal lines selected by litter size at weaning, as demonstrated in line H. PUN tended to increase systematically, with average daily gain being regularly lower with MHL diet.

## 4. Discussion

In the current study, PUN varied depending on the sampling conditions, being widely higher when blood samples were taken at 21:00h—i.e., 3 h after refeeding following a 10-h fasting period—than at 8:00h under ad libitum feeding. These results closely agree with those obtained by [14] for the same times and feeding managements, as a result of low feed intake during morning (when rabbits are practicing caecotrophy) and the overeating at evening after a fasting period—since the higher the protein intake, the higher the PUN [22], because of the catabolism of a greater amount of leftover amino acids. Additionally, the essential amino acid content in microbial proteins recycled through soft feces could improve the quality of dietary protein and reduce protein catabolism, although the amino acid supply from soft feces does not seem to be enough to alter the amino acid pattern of conventional diets [23].

From the results at 21:00h, when there is no caecotrophy interference, the MHL diet seemed to be the candidate to be the best-balanced diet in order to minimize PUN. Although the other three diets (LHM, MMH and LLL diets) induced PUN values that were not significantly higher than the MHL diet, there are some indications in the results obtained at 08:00h that these diets would be suboptimal. Thus, L lysine diets (as LHM and LLL diets) caused higher PUN values at 08:00h; probably because of a lysine deficiency that was not compensated with a supply from soft feces; according to the literature [24,25,26] lysine content in soft feces averages 4.9% of CP, although with some diets this value is lower than in L lysine diets (4.3% of CP). Moreover, increasing threonine level (as in LHM and MMH diet) provoked higher PUN values at 08:00h compared to L threonine diets, which could be interpreted as a result of an excess of threonine; in fact, soft feces are particularly rich in threonine, the only essential amino acid to whose supply they contribute significantly more than to CP supply [22], averaging 5.5% of CP according the above cited literature, and usually being higher than in H threonine diets (5.3% of CP).

In an experiment combining 5 levels of lysine with 3 levels of sulphur amino acids, the minimum urinary nitrogen excretion in rabbits was estimated to be 7.9 and 6.4 g/kg DM for lysine and sulphur amino acids, respectively [27].

On the other hand, PUN at 21:00h increased with average daily gain, probably as a consequence of increasing feed intake. Interestingly, the MHL diet minimized PUN throughout the entire average daily gain range, compared with diets varying only in the level of one amino acid, suggesting that the requirements in the studied amino acids, expressed as a proportion of DM, would not be dependent on growth rate. 

Taking the usual recommendations (established for a diet containing 11.3 MJ DE/kg DM, and then being 0.72, 0.51 and 0.61 g/MJ DE for lysine, sulphur amino acids and threonine, respectively) into account, these results suggest that a diet containing more lysine and sulphur amino acids per energy unit (around 0.82 and 0.67 g/MJ DE) could better fit the growing rabbit requirements, although studies on the effects of such a diet on performance and protein retention are necessary.

## 5. Conclusions

As PUN is a marker of the efficiency of amino acid use, the combination of the three main limiting amino acids minimizing it found in the current study is a good candidate to be tested in order to improve performance and reduce pollution in growing rabbits.

## Figures and Tables

**Table 1:** Ingredients of basal mixture and average chemical composition of the 27 experimental diets.

Ingredients	g/kg	Chemical Composition	g/kg Dry Matter
Wheat bran	300	Dry matter (g/kg^) 2^	907
DDGS corn	50	Ash ^2^	104
Bakery by-product	30	Crude protein ^2^	155
Sunflower meal	36	Crude fat ^2^	29.7
Alfalfa meal	334	Neutral detergent fiber ^2^	455
Beet pulp	80	Acid detergent fiber ^2^	262
Straw	136	Acid detergent lignin ^2^	52.9
		Digestible Energy (Mj/kg DM) ^2^	9.86
Beet molasses	13.85	Amino acid composition ^3^	
L-Arginine	3.1	Aspartic acid	12.95
L-Histidine	1.5	Serine	5.74
Calcium carbonate	6.55	Glutamic acid	22.96
Sodium clorhide	4	Glycine	6.53
Vitamin/Trace	5	Histidine	3.57
element mixture ^1^		Arginine	9.28
		Threonine	VAR ^4^
		Alanine	6.69
		Proline	8.04
		Cystine	2.37
		Tyrosine	2.90
		Valine	7.03
		Methionine	VAR ^4^
		Isoleucine	5.07
		Lysine	VAR ^4^
		Leucine	9.70
		Phenylalanine	5.58

^1^ Contains per kg of feed: vitamin A: 8375 IU; vitamin D3: 750 IU; vitamin E: 20 mg; Vitamin K3: 1 mg; vitamin B1: 1 mg; vitamin B2: 2 mg; vitamin B6: 1 mg; nicotinic acid: 20 mg; choline chloride: 250 mg; magnesium: 290 mg; manganese: 20 mg; zinc: 60 mg; iodine: 1.25 mg; iron: 26 mg; copper: 10 mg; cobalt: 0.7 mg; butyl hydroxylanysole and ethoxiquin mixture: 4 mg.^2^ Provided by the manufacturer (NANTA, Valencia, Spain). ^3^ Analyzed. ^4^ Levels of these amino acids vary depending on experimental diets.

**Table 2:** Levels (g/kg DM) of the three studied amino acids.

Level	Lysine	Sulphur Amino Acids (Cystine + Methionine)	Threonine
High (H)	9.4	6.6 (2.37 + 4.23)	7.8
Medium (M)	8.1	5.8 (2.37 + 3.43)	6.9
Low (L)	6.7	4.9 (2.37 + 2.53)	5.7

**Table 3:** Plasmatic urea nitrogen (PUN) according to sampling time and the dietary level (H: High, M: Medium; L: Low) of lysine (Lys), sulphur amino acids (sAA) and threonine (Thr). Least square means ± standard errors.

Title	08:00h				21:00h			
	H	M	L	*p*-Value	H	M	L	*p*-Value
Lys	10.93 ± 0.136 ^a^	11.18 ± 0.138 ^a^	12.13 ± 0.136 ^b^	<0.001	18.57 ± 0.217 ^b^	17.40 ± 0.217 ^a^	17.63 ± 0.215 ^a^	<0.001
	*n*= 302	*n*= 296	*n* = 304		*n* = 296	*n* = 295	*n* = 298	
sAA	11.44 ± 0.137	11.47 ± 0.135	11.33 ± 0.136	0.759	17.78 ± 0.219	17.56 ± 0.214	18.26 ± 0.216	0.061
	*n* = 301	*n* = 306	*n* = 295		*n* = 288	*n* = 303	*n* = 298	
Thr	11.90 ± 0.136 ^c^	11.36 ± 0.137 ^b^	10.97 ± 0.136 ^a^	<0.001	18.12 ± 0.217	17.94 ± 0.217	17.54 ± 0.214	0.150
	*n*= 304	*n* = 299	*n* = 299		*n* = 293	*n* = 294	*n* = 302	

^a, b, c^ Least square means in the same row with no common superscripts differ significantly at *p* < 0.05.

## Abbreviations

ADG	Average daily gain
CP	Crude protein
DE	Digestible Energy
DM	Dry matter
H	High
L	Low
M	Medium
PUN	Plasmatic urea nitrogen
SAS	Statistical analysis system
